# Advancements in the Management of Endocrine System Disorders and Arrhythmias: A Comprehensive Narrative Review

**DOI:** 10.7759/cureus.46484

**Published:** 2023-10-04

**Authors:** Yogita Kumari, Pooja Bai, Fahad Waqar, Ahmad Talal Asif, Beena Irshad, Sahil Raj, Vaidheesh Varagantiwar, Mahendra Kumar, FNU Neha, Surat Chand, Satesh Kumar, Giustino Varrassi, Mahima Khatri, Tamam Mohamad

**Affiliations:** 1 Medicine, Jinnah Postgraduate Medical Centre, Karachi, PAK; 2 Internal Medicine, Liaquat University of Medical and Health Sciences, Jamshoro, PAK; 3 Medicine, Allama Iqbal Medical College, Lahore, PAK; 4 Medicine, King Edward Medical University (KEMU) Lahore, Lahore, PAK; 5 Medicine, Sharif Medical and Dental College, Lahore, PAK; 6 Internal Medicine, Jinnah Sindh Medical University, Karachi, PAK; 7 Medicine, Rajiv Gandhi Institute of Medical Sciences, Adilabad, IND; 8 Medicine, Sardar Patel Medical College Bikaner India, Bikaner, IND; 9 Medicine, Peoples University of Medical & Health Science for Women, Nawabshah, PAK; 10 Medicine, Ghulam Mohammad Mahar Medical College, Sukkur, PAK; 11 Medicine and Surgery, Shaheed Mohtarma Benazir Bhutto Medical College, Karachi, PAK; 12 Pain Medicine, Paolo Procacci Foundation, Rome, ITA; 13 Medicine and Surgery, Dow University of Health Sciences, Karachi, Karachi, PAK; 14 Cardiovascular Medicine, Wayne State University, Detroit, USA

**Keywords:** interdisciplinary care, telemedicine, personalized medicine, medical advancements, arrhythmia management, endocrine system disorders

## Abstract

In recent years, notable advancements have been made in managing endocrine system disorders and arrhythmias. These advancements have brought about significant changes in healthcare providers' approach towards these complex medical conditions. Endocrine system disorders encompass a diverse range of conditions, including but not limited to diabetes mellitus, thyroid dysfunction, and adrenal disorders. Significant advancements in comprehending the molecular underpinnings of these disorders have laid the foundation for implementing personalized medicine. Advancements in genomic profiling and biomarker identification have facilitated achieving more accurate diagnoses and developing customized treatment plans. Furthermore, the utilization of cutting-edge pharmaceuticals and advanced delivery systems presents a significant advancement in achieving enhanced glycemic control and minimizing adverse effects for individuals afflicted with endocrine disorders. Arrhythmias, characterized by irregular heart rhythms, present a substantial risk to cardiovascular well-being. Innovative strategies for managing arrhythmia encompass catheter-based ablation techniques, wearable cardiac monitoring devices, and predictive algorithms powered by artificial intelligence. These advancements facilitate the early detection, stratification of risks, and implementation of targeted interventions, ultimately leading to improved patient outcomes. Incorporating technology and telemedicine has been instrumental in enhancing the accessibility and continuity of care for individuals diagnosed with endocrine disorders and arrhythmias. The utilization of remote patient monitoring and telehealth consultations enables prompt modifications to treatment regimens and alleviates the need for frequent visits to the clinic. This is particularly significant in light of the current global health crisis. This review highlights the interdisciplinary nature of managing endocrine disorders and arrhythmias, underscoring the significance of collaboration among endocrinologists, cardiologists, electrophysiologists, and other healthcare professionals. Multidisciplinary care teams have enhanced their capabilities to effectively address the intricate relationship between the endocrine and cardiovascular systems. In summary, endocrine system disorders and arrhythmias management have undergone significant advancements due to groundbreaking research, technological advancements, and collaborative healthcare approaches. This narrative review provides a comprehensive overview of the advancements, showcasing their potential to enhance patient care, improve quality of life, and decrease healthcare expenses. Healthcare providers must comprehend and integrate these advancements into their clinical practice to enhance outcomes for individuals with endocrine system disorders and arrhythmias.

## Introduction and background

The complex orchestration of human physiology depends on the seamless interaction of multiple systems, each playing a distinct role in preserving well-being and equilibrium. Among these, the endocrine system and the cardiac conduction system are recognized as two crucial components that coordinate intricate physiological processes. The former regulates the release of hormones, which play a crucial role in various physiological processes such as metabolism, growth, and reproduction [1]. The latter governs the rhythmic pulsation of the heart, guaranteeing the uninterrupted flow of blood, oxygen, and nutrients necessary for sustaining life. However, when these intricately balanced systems experience disruptions, they can result in significant medical complications, leading to conditions commonly referred to as endocrine system disorders and arrhythmias [2]. Endocrine system disorders encompass a wide range of conditions characterized by hormone imbalances. These imbalances can originate from different endocrine glands, including the thyroid, pancreas, adrenal, and pituitary glands. These disorders encompass various conditions, such as diabetes mellitus, thyroid dysfunction, adrenal insufficiency, and polycystic ovary syndrome (PCOS). Although these conditions vary in their presentation and causes, they are connected by a shared factor of hormonal dysregulation that can significantly impact an individual's health and overall well-being [1]. On the other hand, arrhythmias are a category of cardiac rhythm abnormalities that involve irregularities in the heart's electrical signalling system. These irregularities can vary from harmless palpitations to severe conditions like ventricular fibrillation (VF). Some notable arrhythmias include atrial fibrillation (AF), ventricular tachycardia (VT), and bradycardia. While certain arrhythmias may not exhibit symptoms or have a relatively mild impact, others present substantial risks to individuals, potentially resulting in cardiac arrest and sudden death [2].

Managing disorders related to the endocrine system and arrhythmias is of significant clinical importance, given the widespread occurrence and impact of these conditions on global healthcare. In recent years, notable advancements have been made in these disorders' comprehension, identification, and management. These advancements have been driven by progress in medical technology, pharmacotherapy, and clinical research. This comprehensive narrative review examines recent advancements, innovations, and breakthroughs in managing disorders related to the endocrine system and arrhythmias. The prevalence of endocrine system disorders has become a significant global health concern. It is worth mentioning that diabetes mellitus has become a global health issue, as indicated by the World Health Organization (WHO) estimate that 422 million adults were affected by diabetes in the year 2014 [1]. As mentioned above, the figure has consistently increased, imposing a growing strain on healthcare systems and global economies. Diabetes is a medical condition characterized by chronic hyperglycemia caused by impaired insulin secretion or action. This condition is associated with complications, including cardiovascular disease, neuropathy, nephropathy, and retinopathy. The economic implications of diabetes are substantial, as it is estimated that global healthcare spending associated with diabetes will surpass $760 billion by 2045 [2].

Thyroid disorders have a significant global prevalence, impacting approximately 200 million individuals worldwide [3]. The thyroid gland is an essential endocrine system component and critical in regulating metabolism and overall homeostasis. Thyroid dysfunctions, including hypothyroidism, hyperthyroidism, and thyroid nodules, pose considerable clinical challenges. These disorders have the potential to result in a wide range of clinical manifestations, such as fluctuations in weight, disturbances in mood, and disruptions in metabolic function. In addition, conditions such as PCOS have received growing recognition for their significant implications on women's health [2,3]. PCOS is a prevalent endocrine disorder that primarily affects women in their reproductive years. It is distinguished by hormone level disruptions, irregular menstrual cycle irregularities, and the possibility of infertility [4]. In addition to its reproductive implications, PCOS is linked to metabolic disturbances, insulin resistance, and an elevated risk of cardiovascular disease. As a result, PCOS is a complex disorder with substantial clinical and public health implications. Additionally, the domain of arrhythmias poses distinctive challenges within the discipline of cardiology. AF, the most prevalent sustained arrhythmia, has become a notable issue of public health importance. According to estimates, it is projected that by 2030, the United States will experience a prevalence of approximately 14 to 17 million individuals living with AF [5]. AF presents significant morbidity and mortality risks due to its association with an elevated risk of stroke, heart failure, and overall mortality [6]. Ventricular arrhythmias, such as VT and VF, are serious medical conditions that can potentially be life-threatening. If not promptly addressed, these conditions can lead to sudden cardiac death. These cardiac arrhythmias frequently manifest in individuals with pre-existing structural heart disease, presenting a notable clinical obstacle. Moreover, bradycardias, characterized by abnormally slow heart rates, can result in syncope, compromised organ perfusion, and a diminished quality of life. The worldwide incidence of arrhythmias is anticipated to increase due to the ageing of populations and the growing prevalence of risk factors like obesity and hypertension [7]. Therefore, comprehending and effectively managing arrhythmias to prevent cardiovascular events and enhance patient outcomes is paramount.

The interplay between endocrine system disorders and arrhythmias adds complexity to the landscape. Diabetes mellitus, for instance, is not exclusively a metabolic disorder but also a substantial factor in cardiovascular morbidity and mortality [8]. People diagnosed with diabetes are at an increased risk of developing atherosclerosis, hypertension, and coronary artery disease. These conditions can make individuals more susceptible to experiencing arrhythmias. Thyroid disorders have also been linked to the occurrence of arrhythmias. Both hypothyroidism and hyperthyroidism can potentially disrupt cardiac electrophysiology, impacting the heart's normal rhythm [9,10]. The thyroid hormones significantly impact the electrical properties of the heart, and any disruption in their levels can result in the development of arrhythmias. The complex nature of this relationship highlights the importance of adopting a comprehensive and integrated approach to patient care. Healthcare professionals should be able to identify and effectively address the primary endocrine disorder and its potential cardiovascular implications. In the field of cardiology and electrophysiology, medical professionals need to take into account the hormonal environment when assessing and managing arrhythmias.

This extensive narrative review aims to offer healthcare professionals, researchers, and policymakers a thorough comprehension of the latest developments in managing endocrine system disorders and arrhythmias. This review seeks to provide a comprehensive understanding of the dynamic developments in the medical field by analyzing the most recent research findings, clinical trials, and technological advancements. This narrative review examines the recent advancements in diagnostic tools and techniques utilized to precisely identify endocrine system disorders and arrhythmias. Specifically, it explores the development of customized treatment plans tailored to individual patients, taking into account genomic profiling and the identification of relevant biomarkers. This discussion will focus on the various innovative approaches and therapeutic modalities that have emerged in the field of arrhythmia treatment. These include catheter-based ablation techniques, wearable cardiac monitoring devices, and artificial intelligence-driven predictive algorithms. Highlighting the significance of interdisciplinary collaboration within the healthcare field, it is crucial to recognize the value of cooperation among various healthcare professionals, including endocrinologists, cardiologists, and electrophysiologists. This collaboration is essential in effectively addressing the intricate interplay between the endocrine and cardiovascular systems. Please emphasize the effects of these advancements on patient-centered care, quality of life, and healthcare outcomes. This narrative review is a testament to the unwavering commitment to acquiring knowledge and driving innovation in medicine. This statement highlights the significant impact of research, technology, and collaboration in improving patient care and reducing the impact of endocrine system disorders and arrhythmias on individuals and healthcare systems.

## Review

Methodology

This narrative review employs a systematic and comprehensive approach to gather, evaluate, and synthesize relevant literature. 

Literature Search Strategy

A comprehensive search of electronic databases was conducted, including PubMed/MEDLINE, Embase, Scopus, and Google Scholar. These databases were chosen for their comprehensive biomedical and clinical research literature coverage. The search queries were constructed using a combination of medical subject headings (MeSH) terms and keywords. Terms related to endocrine system disorders, arrhythmias, and advancements in their management were used in the search strategy. Sample search terms included "endocrine disorders," "thyroid dysfunction," "arrhythmia," "diabetes management," and "atrial fibrillation treatment." Boolean operators such as "AND" and "OR" were used to refine the search and combine relevant search terms effectively. For instance, "endocrine disorders AND arrhythmia" was used to identify articles relevant to both topics.

Inclusion and Exclusion Criteria 

Articles were included if they were peer-reviewed, published in English, and contained information on recent advancements in managing endocrine system disorders and arrhythmias. Review articles, original research studies, clinical trials, and expert opinion pieces were considered for inclusion. Articles published before the year 2000 were excluded to focus on recent developments.

Data Collection

The initial database search yielded a broad pool of articles. Titles and abstracts of these articles were screened for relevance. Articles not aligning with the review's scope were excluded at this stage. Full-text versions of potentially relevant articles were obtained and thoroughly reviewed. Additional articles not meeting the inclusion criteria or needing more substantial information on recent advancements were excluded during this stage. Pertinent information from the selected papers, including study design, key findings, methodologies, and references to additional relevant sources, was extracted for incorporation into the review.

Synthesis of Information

The information extracted from the selected articles was categorized into thematic areas, including but not limited to diagnostic advancements, personalized medicine, therapeutic innovations, telemedicine, interdisciplinary collaboration, and patient-centered care. A narrative approach was used to synthesize the information obtained from the selected articles. This involved organizing and presenting the findings in a coherent and logical narrative that follows the review's structure. The selected articles were critically appraised for their quality, relevance, and potential bias. Studies with rigorous methodologies, large sample sizes, and clinical significance were given greater weight in the narrative synthesis.

Data Presentation and Reporting

The review is structured to provide a comprehensive overview of advancements in managing endocrine system disorders and arrhythmias. The information is coherently and organized, following the headings and subheadings outlined in the review's structure.

Limitations

It is acknowledged that the review may be subject to publication bias, as it primarily relies on published peer-reviewed articles. Relevant advancements or studies that have not been published or are in non-English languages may not be included. The review focuses on recent advances, potentially excluding valuable historical context or earlier contributions to the field. The study encompasses a wide range of medical specialties, which may introduce variations in the depth and breadth of information available in different areas.

Ethical Considerations

Ethical approval was not required for this narrative review, as it solely relies on publicly available, peer-reviewed literature analysis.

Historical perspective

The historical evolution of our understanding and management of endocrine disorders and arrhythmias has been a fascinating journey marked by significant milestones and breakthroughs. This section will trace the developments in these domains, shedding light on the fundamental discoveries and innovations that have paved the way for modern advancements in the field.

Understanding and Treatment of Endocrine Disorders

The history of endocrinology can be traced back to ancient civilizations where observations of endocrine-related phenomena were made. In ancient Greece, the term "goiter" was coined to describe the enlargement of the thyroid gland. However, its hormonal basis was not understood at the time [11]. Ancient Indian texts also documented the clinical manifestations of diabetes-like symptoms and prescribed treatments. However, the endocrine nature of diabetes was not recognized [12], and the 19th century witnessed significant advancements in understanding endocrine disorders.

In 1850, Thomas Addison described the condition now known as Addison's disease, recognizing the importance of the adrenal glands in maintaining health. Simultaneously, the work of Parry and Graves led to the recognition of Graves' disease and its association with thyroid dysfunction. These discoveries laid the foundation for understanding autoimmune endocrine disorders [13]. The early 20th century marked a watershed moment in endocrinology with the discovery of hormones. In 1905, Bayliss and Starling introduced the concept of hormone signaling with their description of secretin. This hormone regulates pancreatic secretion [14]. This laid the groundwork for understanding hormonal regulation in the endocrine system. One of the most significant breakthroughs in endocrinology occurred with the discovery of insulin.

In 1921, Banting and Best successfully isolated insulin, a hormone produced by the pancreas, leading to a life-saving treatment for diabetes. This marked the birth of modern endocrine therapy and the dawn of personalized medicine for diabetes management [15]. The 20th century saw the development of hormone replacement therapy (HRT) for various endocrine disorders. For instance, the introduction of levothyroxine in the 1950s revolutionized the treatment of hypothyroidism. Additionally, using corticosteroids in the 1940s transformed the management of adrenal insufficiency [15]. The development of diagnostic imaging techniques, such as ultrasound and scintigraphy, in the 20th century greatly enhanced the diagnosis and management of endocrine disorders. These technologies allowed for non-invasive visualization and assessment of endocrine glands, improving the accuracy of diagnoses [16].

Understanding and Treatment of Arrhythmias 

The history of arrhythmia dates back to ancient times when irregular heartbeats were noted by physicians like Hippocrates in ancient Greece. However, the mechanisms behind arrhythmias remained a mystery. In the late 19th century, we witnessed a pivotal moment with the invention of the electrocardiogram (ECG) by Willem Einthoven in 1903. This groundbreaking technology allowed the recording of electrical activity in the heart. It provided valuable insights into the underlying mechanisms of arrhythmias [16]. In the 20th century, the role of the atrioventricular (AV) node in cardiac conduction and bradycardia became clearer. The identification of AV block by Wenckebach in 1899 (Wenckebach, 1899) and Mobitz in 1924 (Mobitz, 1924) marked essential milestones in understanding the electrical conduction system of the heart [16]. The development of cardiac catheterization techniques in the mid-20th century allowed for assessing arrhythmias' structural and electrical causes [17]. This innovation laid the groundwork for more precise diagnostic and therapeutic interventions. The development of antiarrhythmic drugs, such as quinidine and procainamide, in the 20th century, marked a significant advancement in arrhythmia management [18]. These medications could suppress arrhythmias and restore normal cardiac rhythm. Catheter ablation, a technique used to treat arrhythmias by selectively destroying abnormal cardiac tissue, was introduced in the late 20th century [18]. This minimally invasive procedure has revolutionized the treatment of certain arrhythmias. Implantable devices, such as pacemakers and implantable cardioverter-defibrillators (ICDs), became increasingly sophisticated and widely used in the late 20th century [19]. These devices provided life-saving interventions for individuals with bradyarrhythmia and tachyarrhythmias. The 21st century has seen further refinements in catheter ablation techniques and the development of advanced cardiac mapping systems. These advancements have greatly improved arrhythmia treatments' precision and success rates [20]. The historical perspective of endocrine disorders and arrhythmias reveals a fascinating journey from ancient observations to modern advancements. Key milestones, such as the discovery of insulin and the invention of the electrocardiogram, have revolutionized our ability to understand, diagnose, and treat these conditions. These historical developments continue to shape the landscape of endocrinology and cardiology, emphasizing the importance of ongoing research and innovation in improving patient care and outcomes.

Advances in diagnostic tools

Medicine is continually evolving, and nowhere is this more evident than in diagnostics. Advances in diagnostic tools have played a pivotal role in early detection, accurate characterization, and personalized treatment of various medical conditions. This comprehensive discussion will explore the latest diagnostic techniques and technologies in endocrine system disorders and arrhythmias. We will specifically delve into the role of genomic profiling, biomarker identification, and cutting-edge imaging modalities in enhancing our understanding and management of these complex medical conditions.

Genomic Profiling: Unlocking the Genetic Code

Genomic profiling has emerged as a transformative tool in diagnosing and managing endocrine disorders and arrhythmias. It involves the comprehensive analysis of an individual's genetic material to identify specific genetic variations, mutations, or alterations that may predispose them to these conditions. Genomic profiling encompasses next-generation sequencing (NGS), whole-genome, and whole-exome sequencing. Genomic studies have identified specific genetic variants associated with PCOS, shedding light on its underlying genetic basis [21]. These findings offer opportunities for personalized treatment and risk prediction. Genetic markers related to thyroid dysfunction have been identified, allowing for a better understanding of the genetic susceptibility to conditions like Graves' disease and Hashimoto's thyroiditis [22]. Genomic profiling has been instrumental in identifying mutations in ion channel genes responsible for long QT syndrome (LQTS) [23]. This knowledge has led to targeted therapies and risk stratification. Genetic testing has revealed mutations in sodium channel genes as a cause of Brugada syndrome, guiding clinical management [23]. Genomic profiling not only aids in diagnosis but also allows for the development of precision medicine approaches, tailoring treatments to an individual's genetic makeup. Additionally, it enables the identification of at-risk family members, facilitating preventive measures.

Biomarker Identification: Unveiling Disease Signatures

Biomarkers are measurable indicators of normal biological processes, pathogenic processes, or pharmacological responses to therapeutic interventions. In the context of endocrine disorders and arrhythmias, biomarker identification has grown increasingly important as it can enable early disease detection, prognostication, and monitoring of treatment responses. Here, we explore recent advancements in biomarker discovery. Glycated haemoglobin (HbA1c) remains a cornerstone biomarker for diabetes management, but novel biomarkers are being explored. For example, the discovery of specific microRNAs (miRNAs) associated with diabetes has shown promise in early diagnosis and predicting disease progression [23]. Thyroid-stimulating hormone (TSH) and thyroxine (T4) levels have traditionally been used to diagnose thyroid dysfunction. Recent research has focused on identifying novel biomarkers, including serum thyroglobulin and thyroid peroxidase antibodies, to improve diagnostic accuracy [24].

Arrhythmias

Biomarkers like brain natriuretic peptide (BNP) and N-terminal pro-BNP have emerged as predictors of AF recurrence and severity [22]. Additionally, circulating microRNAs have shown potential as diagnostic and prognostic biomarkers in AF. Identifying biomarkers for risk stratification in ventricular arrhythmias is crucial. Biomarkers like C-reactive protein (CRP) and cardiac troponins have been studied for their association with arrhythmic events [23]. Advancements in high-throughput technologies, such as mass spectrometry and proteomics, have accelerated biomarker discovery by allowing the simultaneous analysis of thousands of molecules in biological samples. These emerging biomarkers promise to improve the precision and timeliness of diagnosis in endocrine disorders and arrhythmias.

Cutting-Edge Imaging Modalities: Visualizing Anatomy and Function

In diagnosing and managing endocrine disorders and arrhythmias, imaging modalities have undergone remarkable advancements, enabling clinicians to visualize anatomical structures and functional aspects of the endocrine and cardiovascular systems with unprecedented detail. High-resolution ultrasound has become the standard for evaluating thyroid nodules, aiding in the characterization of nodules as benign or suspicious for malignancy [24]. Positron emission tomography (PET) imaging with radiolabeled tracers, such as 18F-fluorodeoxyglucose (FDG), has proven valuable in detecting neuroendocrine tumors [25]. Advances in ECG technology, including 12-lead Holter monitors and mobile ECG devices, have improved the detection and monitoring of arrhythmias, especially AF [26]. Cardiac MRI provides exquisite anatomical and functional information, aiding in assessing arrhythmia substrates, myocardial infarctions, and cardiomyopathies. Advances in diagnostic tools have ushered in a new era of precision medicine, revolutionizing the diagnosis and management of endocrine disorders and arrhythmias. Genomic profiling has unveiled the genetic underpinnings of these conditions, paving the way for personalized treatment approaches. Biomarker identification offers promising avenues for early diagnosis and treatment monitoring. Cutting-edge imaging modalities provide clinicians with unprecedented insights into the anatomy and function of the endocrine and cardiovascular systems. These advancements enhance diagnostic accuracy and transform healthcare delivery, allowing for tailored interventions that improve patient outcomes and quality of life. As research evolves, the future promises even more sophisticated and effective diagnostic tools for better healthcare.

Personalized medicine in endocrine disorders

In recent years, personalized medicine has emerged as a groundbreaking paradigm in healthcare, revolutionizing the diagnosis and treatment of various medical conditions, including endocrine disorders. This approach acknowledges the inherent variability among individuals and seeks to tailor medical interventions to a patient's unique genetic makeup, lifestyle, and clinical characteristics. In this comprehensive discussion, we will explore how advancements in personalized medicine are reshaping the management of endocrine disorders. We will delve into the role of genomic data, individualized therapy plans, and the tangible improvements in patient outcomes resulting from this patient-centric approach.

The Era of Genomic Medicine: Unraveling Genetic Complexity

Genomic medicine has been a pivotal driver of personalized treatment in endocrine disorders. The human genome project, completed in 2003, marked a significant milestone by providing a comprehensive map of the human genome's structure and function. This monumental achievement paved the way for a deeper understanding of the genetic underpinnings of endocrine disorders, enabling clinicians to unravel their complexities and devise targeted therapies. PCOS, a common endocrine disorder affecting women, showcases the power of genomic medicine. Recent studies have identified specific genetic variants associated with PCOS, shedding light on its multifactorial aetiology [27]. This genetic insight allows for tailored treatment approaches based on an individual's genetic predisposition, potentially optimizing therapeutic outcomes. The genetic basis of thyroid disorders, including autoimmune conditions like Graves' disease and Hashimoto's thyroiditis, has been elucidated through genomic research [27]. Understanding the genetic susceptibility of patients can guide therapeutic choices, such as the selection of antithyroid drugs or the consideration of thyroidectomy.

Tailored Therapy Plans: Precision in Action

Advancements in genomics have paved the way for the development of individualized therapy plans, where treatment decisions are guided by a patient's unique genetic profile, biomarker data, and clinical presentation. These personalized approaches offer tangible benefits, including improved treatment efficacy and minimizing adverse effects. Diabetes management exemplifies the shift toward personalized care. Genetic testing can identify monogenic forms of diabetes, which differ significantly from the more common type 2 diabetes [28]. Accurate classification of diabetes subtypes informs treatment strategies, such as sulfonylureas for patients with monogenic diabetes. In the realm of adrenal disorders, personalized approaches have gained traction. For instance, patients with primary aldosteronism may undergo adrenal vein sampling to localize the source of excess aldosterone production [28]. This information guides therapeutic decisions, such as deciding between surgery and medical management.

Genomic Data in Decision-Making: Realizing Improved Outcomes

Integrating genomic data into clinical decision-making has yielded substantial benefits in terms of patient outcomes. By tailoring treatments to an individual's genetic makeup, healthcare providers can optimize therapeutic responses, minimize adverse effects, and enhance overall quality of life. In cancer-related endocrine disorders like thyroid cancer, genomic testing can identify specific genetic mutations (e.g., BRAF, RET) that inform prognosis and guide treatment choices [29]. Targeted therapies, including tyrosine kinase inhibitors, have emerged as effective treatments for patients with specific genetic alterations. Genomic analysis has revealed the genetic basis of growth hormone deficiency, allowing for the identification of gene mutations responsible for this condition [30]. This genetic insight enables the precise prescription of growth hormone replacement therapy, leading to improved growth and quality of life for affected individuals.

Challenges and Considerations

The handling of genomic data raises concerns about privacy and ethical implications. Safeguarding patient information and ensuring informed consent are critical considerations in personalized medicine [31]. Genomic testing can be expensive, and access to these services may be limited in some regions. Efforts to make genomic testing more cost-effective and accessible are ongoing [23]. Successful implementation of personalized medicine often requires collaboration among healthcare professionals from various specialties, including genetic counsellors, endocrinologists, and pharmacists [32]. Patients need to be informed about the benefits and limitations of genomic testing. Clear communication and education are essential to ensure patients can make informed decisions about their healthcare [33]. Personalized medicine has ushered in a new era of patient-centric care, transforming the landscape of endocrine disorder management. Genomic data and individualized therapy plans are at the forefront of this revolution, allowing clinicians to tailor treatments to each patient's unique genetic and clinical characteristics. The result is improved treatment efficacy, minimized adverse effects, and enhanced patient outcomes. As genomic research advances, the potential for personalized medicine to improve the lives of individuals with endocrine disorders remains promising. However, addressing challenges related to data privacy, cost, interdisciplinary collaboration, and patient education will be crucial in realizing the full potential of personalized medicine in endocrinology.

Innovations in arrhythmia management

Arrhythmias, irregular heart rhythms, have long been a challenge in cardiology. Over the years, innovations in arrhythmia management have transformed the landscape of diagnosis and treatment, offering new hope for patients with these conditions. This comprehensive discussion will delve into cutting-edge methods for diagnosing and treating arrhythmias. Specifically, we will explore catheter-based ablation techniques, cardiac monitoring devices, and predictive algorithms, highlighting how these innovations revolutionize arrhythmia management. Catheter-based ablation techniques have emerged as a cornerstone of arrhythmia management, offering precise and effective treatment options for patients with various arrhythmic disorders. These techniques involve using catheters to target and eliminate the abnormal electrical pathways responsible for arrhythmias. AF, the most common sustained arrhythmia, has witnessed significant advancements in ablation strategies. Pulmonary vein isolation (PVI) has become a standard approach, targeting triggers in the pulmonary veins [34]. Recent innovations include using advanced mapping technologies like contact force sensing catheters, enhancing the accuracy of lesion delivery. Catheter-based ablation has also revolutionized VT management. Substrate mapping, involving identifying scar tissue and abnormal electrograms, plays a crucial role in VT ablation [35]. Stereotactic body radiation therapy (SBRT) has emerged as a novel non-invasive approach for refractory VT [36]. SVTs, including atrioventricular nodal reentrant tachycardia (AVNRT) and atrioventricular reentrant tachycardia (AVRT), are amenable to catheter ablation. Radiofrequency ablation at specific anatomical sites, such as the slow pathway for AVNRT, has high success rates. These catheter-based approaches have improved the efficacy of arrhythmia treatment and reduced complications, shorter hospital stays, and enhanced patient outcomes.

Cardiac Monitoring Devices: Continuous Vigilance

Cardiac monitoring devices have evolved to provide continuous, real-time arrhythmias surveillance, aiding in diagnosis and treatment. These devices come in various forms, from wearable monitors to implantable devices, enabling healthcare providers to capture crucial data for informed decision-making. ICDs have become a standard of care for patients at risk of sudden cardiac death due to ventricular arrhythmias [37]. Advanced ICDs provide defibrillation and offer continuous monitoring and remote data transmission to healthcare providers. Devices like pacemakers and implantable loop recorders (ILRs) have seen remarkable advancements. ILRs, in particular, have become powerful tools for diagnosing cryptogenic strokes and unexplained syncope by recording heart rhythm data over extended periods [37]. The proliferation of wearable technology, including smartwatches and chest patches, has democratized cardiac monitoring. These devices can detect arrhythmias, record ECGs, and provide real-time alerts to users and healthcare professionals [38].

Predictive Algorithms: Anticipating Arrhythmias

Integrating artificial intelligence (AI) and predictive algorithms into arrhythmia management has ushered in a new era of proactive care. These algorithms leverage vast datasets to predict arrhythmic events, enabling early intervention and prevention. AI-based predictive algorithms analyze patients' ECG data to forecast AF episodes. Such algorithms can aid in the timely initiation of anticoagulation therapy to prevent stroke. Predictive models for VT and VF can identify patients at high risk of sudden cardiac death [39]. These algorithms inform the selection of appropriate therapies, including ICD implantation. Predictive algorithms can be seamlessly integrated into telehealth platforms, allowing remote monitoring of high-risk patients. This approach ensures timely intervention and reduces healthcare system burden. Using cardiac monitoring devices and predictive algorithms raises data security and privacy concerns. Ensuring the secure transmission and storage of patient data is paramount. Access to advanced arrhythmia management tools should be equitable across diverse populations. Disparities in access to care and technology must be addressed. Predictive algorithms require rigorous validation to ensure their accuracy and clinical utility. Collaboration between clinicians and data scientists is essential in this regard. Balancing the cost of implementing these innovations with their clinical benefits is crucial. Health economic analyses are essential to determine cost-effectiveness. Innovations in arrhythmia management are ushering in a new era of precision and proactive care. Catheter-based ablation techniques are offering effective and less invasive treatment options. Cardiac monitoring devices are providing continuous vigilance, enabling early diagnosis and intervention. Predictive algorithms are helping anticipate arrhythmic events, potentially saving lives. As technology advances and research in this field progresses, arrhythmia management will become even more patient-centric and data-driven. Addressing challenges related to data security, health equity, clinical validation, and cost-effectiveness will be crucial in harnessing the full potential of these innovations to improve the lives of individuals with arrhythmias.

Telemedicine and remote patient monitoring

Telemedicine and remote patient monitoring have revolutionized healthcare by transcending geographical boundaries and enabling patients to access high-quality care from their homes. This transformation has significant implications for the management of endocrine disorders and arrhythmias. In this comprehensive analysis, we will explore the role of telemedicine and remote monitoring in these two medical domains, emphasizing how these technologies enhance access to care, promote patient compliance, and contribute to improved health outcomes.

Telemedicine in Endocrine Management

Endocrine disorders, which encompass conditions affecting the thyroid, adrenal glands, and pancreas, often require ongoing monitoring and medication adjustments. Telemedicine enables endocrinologists to conduct remote consultations with patients, eliminating the need for frequent in-person visits. This is particularly beneficial for individuals with conditions like diabetes, where regular check-ups are essential. Integrated with telemedicine platforms, CGM devices allow patients to transmit real-time glucose data to their healthcare providers. This facilitates timely adjustments to insulin regimens and improves glycemic control [40]. Patients can receive medication management and dosage adjustments through telemedicine, reducing non-adherence risk. Telehealth visits also enhance patient education about the importance of medication adherence [40].

Remote Monitoring in Arrhythmia Management

Arrhythmias, including AF and VT, require continuous monitoring and prompt intervention. These portable devices can record cardiac rhythms over extended periods and transmit data remotely. Patients with suspected arrhythmias can wear these devices, and healthcare providers can review the recorded data remotely, leading to early diagnosis and treatment. Implantable devices such as pacemakers and defibrillators have remote monitoring capabilities. These devices automatically transmit data to healthcare providers, allowing them to detect and address arrhythmias promptly [24]. Specialized arrhythmia clinics offer telehealth appointments for arrhythmia patients. These clinics provide expert consultation and remote monitoring, ensuring patients receive the necessary care and support.

Improving Access to Care

Patients in rural areas often face challenges in accessing specialized endocrine or cardiology care. Telemedicine bridges this gap, enabling remote consultations with specialists. Patients with chronic endocrine disorders or arrhythmias may need frequent follow-up visits. Telemedicine eliminates the need for extensive travel, reducing patients' financial and logistical burdens. Telemedicine allows patients to consult with renowned specialists worldwide, breaking geographical barriers. This is particularly advantageous for individuals with complex or rare conditions [41].

Enhancing Patient Compliance

Remote patient monitoring encourages patients to adhere to regular check-ups and monitoring. Knowing that their data is being transmitted and reviewed by healthcare providers incentivizes patients to stay engaged in their care. Telehealth visits offer opportunities for healthcare providers to discuss medication adherence with patients. Medication regimens can be adjusted based on patient feedback and monitoring data. Telehealth appointments provide a platform for patient education. Healthcare providers can educate patients about lifestyle modifications, medication compliance, and self-monitoring, leading to better adherence [26].

Challenges and Considerations

Patients may need access to the necessary technology or have limited digital literacy. Efforts are needed to ensure equitable access. Protecting patient data from breaches and ensuring compliance with privacy regulations is paramount. Policies and reimbursement models must evolve to support telehealth and remote monitoring, ensuring financial sustainability. Seamless integration of telemedicine and remote monitoring with electronic health records (EHRs) is essential to streamline care coordination [27]. Telemedicine and remote patient monitoring have emerged as transformative tools in managing endocrine disorders and arrhythmias. These technologies improve access to specialized care, enhance patient compliance, and improve health outcomes. Healthcare systems can harness the full potential of telemedicine and remote monitoring to deliver patient-centred care by addressing technology access, data security, reimbursement, and interoperability challenges. In a rapidly evolving healthcare landscape, telemedicine and remote monitoring continue to shape the future of healthcare delivery, offering innovative solutions for patients with chronic and complex conditions.

Interdisciplinary collaboration

The demand for comprehensive and patient-centred care has become increasingly imperative in the rapidly evolving healthcare landscape. To meet this demand, interdisciplinary collaboration among healthcare professionals has emerged as a fundamental approach. Collaboration among healthcare specialists enhances the quality of care. It improves patient outcomes, particularly in complex fields such as endocrinology and cardiology. This comprehensive essay emphasizes the importance of interdisciplinary collaboration. It shows how endocrinologists, cardiologists, and other specialists work together to provide holistic care. By exploring how these professionals collaborate and sharing pertinent studies and evidence, this essay will underscore the vital role of interdisciplinary collaboration in modern healthcare.

Importance of Interdisciplinary Collaboration

Interdisciplinary collaboration in healthcare is the practice of healthcare professionals from various fields working together to provide patient-centred care. This collaboration extends beyond the conventional silos of medical specialties and fosters a comprehensive, holistic approach to patient management. Collaboration allows healthcare providers to address all aspects of a patient's health, considering physical and psychological factors. This comprehensive approach leads to a more accurate diagnosis and effective treatment plan. Research consistently demonstrates that interdisciplinary collaboration results in improved patient outcomes. A study published in the Journal of General Internal Medicine found that collaborative care models for chronic diseases led to better clinical outcomes and increased patient satisfaction [29]. Collaboration can lead to more efficient use of resources, reducing redundancy and unnecessary tests. This, in turn, lowers healthcare costs while maintaining or improving the quality of care. Interdisciplinary teams bring together diverse perspectives, allowing for more creative problem-solving and innovative approaches to patient care [31]. Collaboration emphasizes patient preferences and values, ensuring the care plan aligns with the patient's goals and needs.

Interdisciplinary Collaboration in Endocrinology

Endocrinology is a medical specialty focused on the endocrine system, encompassing hormone-producing glands and their hormones. Conditions treated by endocrinologists include diabetes, thyroid disorders, and hormonal imbalances. Collaborative care in endocrinology involves working closely with other specialists to address the diverse aspects of patients' health. Diabetic patients often require a multi-faceted approach involving endocrinologists, dietitians, and primary care physicians. For instance, a study in Diabetes Care highlighted the benefits of a team-based care approach involving endocrinologists and pharmacists in managing type 2 diabetes, leading to improved glycemic control [32]. Patients with thyroid disorders may need the expertise of an endocrinologist in conjunction with a surgeon if surgery is required or a radiologist for thyroid imaging. This collaborative approach ensures a thorough evaluation and tailored treatment. Endocrinologists collaborate with mental health professionals when hormonal imbalances lead to mood disorders. Such collaboration helps address both the physical and psychological aspects of the condition.

Interdisciplinary Collaboration in Cardiology

Cardiology focuses on the heart and circulatory system, addressing conditions like heart disease, arrhythmias, and congestive heart failure. Collaboration in cardiology is crucial for a holistic understanding and management of cardiovascular diseases. Cardiologists work closely with primary care physicians to assess cardiovascular risk factors such as hypertension, high cholesterol, and diabetes. This collaboration enables early intervention and lifestyle modifications. In cases where invasive procedures like angioplasty or stent placement are necessary, cardiologists collaborate with interventional radiologists or cardiovascular surgeons. This multidisciplinary approach ensures that the best course of action is taken. The management of congestive heart failure often involves a team of healthcare professionals, including cardiologists, nurses, pharmacists, and dietitians. Collaboration helps in optimizing medication regimens and educating patients on self-care.

The Collaborative Model

One can examine the Collaborative Care Model, often employed in addressing chronic illnesses, to understand how interdisciplinary collaboration operates in healthcare. This model is particularly relevant in the context of endocrinology and cardiology. Healthcare providers from various disciplines, including physicians, nurses, psychologists, social workers, and pharmacists, work together to provide patient-centered care. A designated care coordinator ensures that information is shared among team members and that the patient's care plan is cohesive and consistent. Team members meet regularly and communicate to discuss patient progress, adjust treatment plans, and address any challenges or concerns. Patients are actively involved in their care and decision-making, with clear communication and education provided to help them manage their condition effectively. A study in JAMA Internal Medicine demonstrated the effectiveness of the Collaborative Care Model in improving outcomes for patients with depression and chronic medical conditions [34]. This model can easily be adapted to address the complex needs of patients with endocrine and cardiac disorders. Interdisciplinary collaboration among healthcare professionals is paramount in providing holistic and patient-centered care. In endocrinology and cardiology, collaboration between specialists leads to improved patient outcomes, enhanced problem-solving, efficient resource utilization, and better healthcare delivery. The Collaborative Care Model exemplifies how interdisciplinary teams can work together to address complex health conditions effectively. By emphasizing the importance of interdisciplinary collaboration and showcasing its practical application in patient care, healthcare systems can better meet the evolving needs of patients in today's dynamic healthcare landscape.

Patient-centered care

Patient-centred care is a fundamental principle of modern healthcare that places the patient at the forefront of decision-making and treatment. It encompasses the physical aspects of medical treatment and healthcare's emotional, psychological, and social dimensions. Over the years, healthcare delivery and management advancements have significantly transformed the patient experience and quality of life. This essay explores the impact of these advancements on patient experience and quality of life, drawing on patient testimonials and case studies to illustrate the benefits of modern management approaches.

Advancements in Patient-Centered Care

The integration of technology has revolutionized healthcare. EHRs, telemedicine, wearable devices, and mobile health apps have enhanced patient engagement and communication with healthcare providers. Patients can now access their medical records, schedule appointments, and receive real-time health data, empowering them to participate in their care actively [36]. Advances in genomics and precision medicine have allowed for tailored treatment plans based on an individual's genetic makeup. Personalized medicine ensures that treatments are more effective and have fewer side effects, resulting in a higher quality of life for patients [37]. Access to reliable health information online has enabled patients to become more informed about their conditions and treatment options. Educational resources, such as online videos and forums, help patients make informed decisions and actively engage in their healthcare journey. Collaborative care models involving multiple healthcare professionals from different disciplines have become more common. This approach ensures that patients receive comprehensive care that addresses their physical, emotional, and social needs. For example, a Journal of General Internal Medicine study found that collaborative care for chronic diseases improved clinical outcomes and patient satisfaction [38].

Impact on Patient Experience

Patients are now more empowered to engage in their healthcare decisions actively. They can access their medical information and communicate with healthcare providers remotely. This enhanced sense of control and involvement leads to better patient experiences. Telemedicine and online appointment scheduling have significantly reduced wait times for routine consultations. Patients no longer have to endure long waits in waiting rooms, leading to increased convenience and satisfaction. Technology-driven communication tools enable patients to ask questions, seek clarifications, and share concerns with their healthcare providers easily. Timely and effective communication enhances trust and patient-provider relationships. Personalized medicine and access to health information allow patients to make informed decisions about their treatment options. They can actively participate in selecting the most suitable treatment plan, resulting in a sense of empowerment [39].

Impact on Quality of Life

Personalized medicine minimizes the risk of adverse reactions to medications, improving the quality of life for patients with chronic conditions. For example, targeted therapies are associated with fewer side effects in oncology than traditional chemotherapy. Telemedicine and mobile health apps enable patients to access healthcare services from the comfort of their homes. This convenience is especially valuable for individuals with mobility limitations or those living in remote areas [40]. Remote monitoring and wearable devices allow healthcare providers to track patients' health parameters continuously. This real-time data helps in early detection and prompt intervention, improving symptom management and overall well-being [40]. Advancements in patient-centered care, driven by technology, personalized medicine, patient education, and collaborative care models, have transformed the healthcare landscape. These advancements have empowered patients, improved their experiences, and enhanced their quality of life. Patient testimonials and case studies highlight the real-world impact of these innovations, demonstrating how modern management approaches are shaping a more patient-centric healthcare system. As healthcare continues to evolve, it is crucial to prioritize patient-centered care to ensure that patients receive adequate treatment and have a positive and empowering healthcare experience.

Challenges and future directions

Endocrinology and arrhythmia management have significantly improved patient care and outcomes. However, they also face significant challenges and limitations that must be addressed to advance patient care further. This essay will explore the challenges and limitations of endocrine and arrhythmia management and speculate on future trends and research directions. By examining the ongoing issues and emerging possibilities, we can gain insight into the evolving healthcare landscape in these critical areas.

Challenges in Endocrine Management

The intricate regulatory mechanisms of hormones make diagnosing and treating endocrine disorders challenging. Hormonal imbalances may have subtle symptoms and require meticulous evaluation. For some endocrine conditions, such as rare genetic disorders, treatment options are limited, and research into new therapies is scarce. Patients with these conditions often face lifelong management challenges. Access to specialized endocrinologists can be limited, especially in rural areas. This geographical disparity can result in delayed diagnosis and management. Hormone replacement therapies, commonly used in endocrine disorders like hypothyroidism, can be costly and financially burden patients, affecting their treatment adherence.

Challenges in Arrhythmia Management

Arrhythmias, especially asymptomatic ones, often go undiagnosed. Routine screenings and monitoring are not standard, sometimes leading to delayed intervention. Some treatment modalities, like antiarrhythmic medications, can have side effects and may not be effective in all patients. Balancing the benefits and risks of treatment is a complex process. Access to electrophysiologists specializing in arrhythmia management can be limited in certain regions, leading to disparities in care quality. Accurate risk stratification for arrhythmias remains challenging, making it difficult to identify high-risk patients who may benefit from interventions like implantable devices.

Future Directions in Endocrine Management

Advances in genomics are leading to personalized treatment plans for endocrine disorders. Tailored therapies based on an individual's genetic profile can optimize treatment efficacy and minimize side effects. Telemedicine can bridge the gap in access to specialized endocrinologists, enabling remote consultations and monitoring for patients in underserved areas. Continuous remote monitoring can enhance disease management. AI-powered tools can assist in the early detection and diagnosis of endocrine disorders by analyzing large datasets and identifying subtle patterns. These technologies can improve diagnostic accuracy. Empowering patients with knowledge about their condition through digital health resources can promote self-management and adherence to treatment plans, reducing the burden on healthcare systems [41].

Future Directions in Arrhythmia Management

The development of non-invasive diagnostic tools, such as wearable ECG monitors and implantable loop recorders, can aid in the early detection of arrhythmias and improve diagnostic accuracy. Ongoing research in electrophysiology is exploring new treatments, such as ablation techniques, that target arrhythmia sources more precisely, resulting in better outcomes and fewer side effects. AI-driven algorithms can analyze patient data to predict arrhythmia risk more accurately. This can aid in identifying high-risk patients who may benefit from preventive interventions. Telehealth solutions are becoming increasingly crucial for arrhythmia management. They enable remote consultations, monitoring, and follow-ups, improving access to specialized care [42]. Endocrine and arrhythmia management are crucial aspects of healthcare, but they face several challenges that impact patient care. These challenges include complex disease mechanisms, limited treatment options, and disparities in access to specialized care. However, the future holds promising developments in both fields. Precision medicine, telemedicine, AI applications, and patient empowerment transform endocrine management. Advanced diagnostic tools, electrophysiology research, AI for risk prediction, and telehealth solutions are shaping the future of arrhythmia management. Healthcare providers and researchers can improve patient outcomes and enhance the quality of care in endocrinology and arrhythmia management by addressing current challenges and focusing on these emerging trends.

## Conclusions

From integrating technology and telemedicine to personalized medicine and artificial intelligence, the healthcare field is experiencing rapid transformation. These advancements can significantly improve patient care, outcomes, and overall quality of life. One overarching theme from this discussion is the importance of staying current with these advancements in clinical practice. Healthcare professionals are entrusted with the well-being of their patients, and by embracing these innovations, they can provide more precise, accessible, and patient-centred care. Telemedicine allows for remote consultations and monitoring, ensuring that even patients in underserved areas can access specialized care. Personalized medicine tailors treatments to individual genetic profiles, minimizing side effects and optimizing therapeutic outcomes. Artificial intelligence assists in early diagnosis, risk prediction, and treatment planning, enhancing accuracy and efficiency. In an era of rapid scientific progress, healthcare providers must continually educate themselves and adapt their practices to incorporate these advancements. By doing so, they improve patient care and contribute to the ongoing evolution of healthcare towards a more patient-centric and effective system. In this dynamic landscape, staying informed and embracing innovation is not just a choice but a responsibility for healthcare professionals dedicated to providing the best possible care to their patients.
